# Risk factors for comorbid epilepsy in patients with psychogenic non-epileptic seizures. Dataset of a large cohort study

**DOI:** 10.1016/j.dib.2022.108568

**Published:** 2022-09-03

**Authors:** Andreu Massot-Tarrús, Yeyao Joe Yu, Mashael AlKhateeb, Seyed M. Mirsattari

**Affiliations:** a Department of Neurology, F. Ass. Mutua Terrassa, Terrassa, Spain; b Department of Clinical Neurological Sciences, Londn, Ontario, Canada; c Medical Biophysics, London, Ontario, Canada; d Medical Imaging, London, Ontario, Canada; e Psychology, Western University, London, Ontario, Canada; f Neurology Section, Department of Neurosciences, King Faisal Specialist Hospital & Research Centre, Riyadh, Saudi Arabia

**Keywords:** Psychogenic nonepileptic attacks, dissociative seizures, functional seizures, pseudoseizures, functional neurological disorder, conversive disorder, differential diagnosis, video-EEG

## Abstract

Psychogenic non-epileptic seizures (PNES) are the main differential diagnosis of pharmacorresistant epilepsy. Achieving the certainty in the diagnosis of PNES may be challenging, especially in the 10-22% of cases in which PNES and epilepsy co-exist. This difficulty hampers the management of these patients. Unfortunately, published series with this combined pathology are scarce and small in size.

This article presents the dataset of our article “Factors associated with comorbid epilepsy in patients with psychogenic non-epileptic seizures: a large cohort study” (Massot-Tarrús et al. 2022). It is composed by a detailed demographic and clinical data of 271 consecutive patients diagnosed with PNES in our epilepsy monitoring unit (EMU) between May 2001 and February 2011, and followed until September 2016. Based on the clinical, neuroimaging and vEEG findings, 47 of these patients were diagnosed with definite comorbid epilepsy, and 30 with possible or probable comorbid epilepsy. All data was collected retrospectively from chart review.

The cohort is depicted by means of demographic variables; age at PNES onset; years with PNES; frequency of PNES; duration of longest PNES seizure; self-reported history of minor head trauma (not associated with an increased risk of epilepsy) immediately preceding the first PNES; history of substance abuse; past or present history of active suicidal ideation; neuropsychological evaluation with the Minnesota Multiphasic Personality Inventory test; number and nature of risk factors for epilepsy; co-morbid degenerative brain disease or other neurological or psychiatric medical conditions; semiology of the seizures and triggers; EEG findings; type of epilepsy; number of past EMU admissions and epilepsy clinic visits and re-referrals; number of Anti-Seizure Medications (ASM) at EMU admission and discharge; and the outcome of the spells and ASM after the EMU discharge. Those ASM prescribed for reasons other than the treatment of the seizures (e.g., psychiatric disorders, migraine, pain syndromes, etc.) were not counted.

The presented baseline data can be used in studies evaluating the characteristics of patients with PNES and comorbid epilepsy, and in the creation of algorithms to identify them. It could facilitate the prioritization of this subgroup of patients for prolonged video-EEG monitorization to confirm the co-existence of both types of seizures and treat them accordingly.

**Specifications Table**

**Table:** 

Subject	Clinical Neurology
Specific subject area	Risk factors associated with the coexistence of epilepsy in patients with psychogenic non-epileptic seizures.
Type of data	Table
How the data were acquired	All was acquired retrospectively from a comprehensive chart review at the London Health Sciences Centre (LHSC, London, Canada) performed by two neurologists with expertise in the field of epilepsy (Y.J.Y. and M.A.), covering the period preceding the diagnosis of PNES until either the last follow-up visit or September 2016, whichever came first, to ensure that the final diagnosis was not revised. A predetermined table with the demographic and clinical variables of interest was created in an excel file to complete.
Data format	Raw
Description of data collection	We enrolled consecutive patients admitted to the EMU at LHSC. Patients were included if EMU admission documented a new diagnosis of PNES based on two habitual seizures or single prolonged psychogenic seizure (lasting more than 30 mins.) captured after a prolonged video EEG monitoring. Two seizures were required for added certainty because PNES have a higher rate of variability in appearance compared to the ES.
Data source location	Institution: London Health Sciences Centre (LHSC, London, Canada). City/Region: London, Ontario. Country: Canada.
Data accessibility	Data identification number: DOI: 10.17632/gmwkgkkyk6.3 Direct URL to data: https://data.mendeley.com/datasets/gmwkgkkyk6/3
Related research article	A. Massot-Tarrús, Y. Joe Yu, M. AlKhateeb, S.M. Mirsattari. Factors associated with comorbid epilepsy in patients with psychogenic nonepileptic seizures: A large cohort study, Epilepsy Behav. 23 (2022) 108780. https://doi.org/10.1016/j.yebeh.2022.108780.

## Value of the Data

• This dataset provides the demographic and clinical characterization of a large cohort of patients with PNES alone or with comorbid epilepsy. The latest is a subgroup of patients with scarce information available in the literature.
• This dataset could be valuable to researchers considering future comparative studies of patients with PNES vs epilepsy alone, or patients with PNES vs PNES and comorbid epilepsy.
• This data can be used for the development of algorithms to identify patients with PNES at higher risk of suffering comorbid epilepsy. Such algorithms might improve the cost-effectiveness of prolonged video-EEG monitorization, shortening the time to diagnosis, and avoiding unnecessary treatments.

## 1 Data Description

Table 1 shows the raw dataset of patients diagnosed with Psychogenic non-epileptic seizures (PNES) in our Epilepsy Monitoring Unit (EMU) between May 2001 and February 2011, and their follow-up until the last follow-up visit or September 2016. It includes patients with or without comorbid epilepsy [1].

The following demographic and clinical variables are included in the table: gender, age at the EMU admission, number of children, marital status, maximum education degree, present and past employment status, valid or suspended driving license and reason, age at PNES onset and years with PNES, frequency of PNES at the time of EMU admission, duration of longest PNES seizure according to either patient or witness (whichever was considered more accurate), report of minor head trauma - stated as a concussion or lesser injury that should not cause an increased risk of epilepsy- by the patient as immediately preceding the initial PNES, neuropsychological evaluation (with the Minnesota Multiphasic Personality Inventory test) results, family history of epilepsy, personal history of febrile seizures, developmental disabilities, meningitis/meningoencephalitis, traumatic brain injury – as amnesia, alteration or loss of consciousness or neurologic deficits after a blow to head [2, 3] - brain lesions on neuroimaging considered of risk for epilepsy, history of substance abuse, dementia (Alzheimer's disease or any other), other neurological or psychiatric medical conditions, history of active suicidal ideation, description of the seizures signs and symptoms, their auras and triggers, EEG findings, type of epilepsy if associated, number of Anti-Seizure Medications (ASM) at EMU admission and discharge, and the outcome of the spells and ASM intake during the follow-up after the EMU discharge (only ASM prescribed for the treatment of the seizures where counted).

The categories in which each variable was divided are described in the headline of the columns.

The definitions, coding and calculation of the variables are described in a second sheet in the same excel file.

## 2 Experimental Design, Materials and Methods

The design of the study is retrospective case-control.

Enrolment of patients was done at the time of their admission to the EMU of the London Health Sciences Centre (LHSC, London, Ontario, Canada), between May 2001 and February 2011. The inclusion criteria were the diagnosis of PNES based on the recording of two usual spells or a single prolonged spell (lasting 30 minutes or more) captured during the video EEG monitorization. The diagnosis was made in all cases by a certified epileptologist and electroencephalographer for the Canadian Society of Clinical Neurophysiologists, meeting therefore the highest level of diagnostic certainly (“Documented PNES”) according to the International League Against Epilepsy (ILAE) [4]. Two seizures were required to reinforce the certainty of the diagnosis, since PNES have a higher rate of inconsistency compared to epileptic seizures.

A predetermined excel table with the defined demographic and clinical variables of interest was created. The table was filled by two neurologists with proficiency in the field of epilepsy (Y.J.Y. and M.A.). In order to do this, the two neurologists performed a comprehensive retrospective chart review of each patient (including clinic notes, discharge summaries, psychology reports and other documents dictated right after the patient encounters) covering the period preceding the diagnosis of PNES until either September 2016 or the last follow-up visit, whichever came first. 235/271 patients had follow-up visits (136 with a short-term follow-up and 120 with a long-term follow-up timepoint).

## Ethics Statements

“Psychogenic non-epileptic seizures (PNESs) REB 100155” from The Health Sciences Research Ethics Board (HSREB) at Western University.

Due to the retrospective design of the study, patient consent was waved by the HSREB.

## CRediT authorship contribution statement

**Andreu Massot-Tarrús:** Formal analysis, Visualization, Methodology, Writing – original draft, Writing – review & editing. **Yeyao Joe Yu:** Data curation, Validation, Investigation, Writing – review & editing. **Mashael AlKhateeb:** Data curation. **Seyed M. Mirsattari:** Conceptualization, Project administration, Resources, Funding acquisition, Supervision, Writing – review & editing.

## Declaration of Competing Interest

The authors declare the following financial interests/personal relationships which may be considered as potential competing interests:

A. Massot-Tarrús has received honoraria for speaking engagements and advisory boards from Bial, Eisai and UCB Pharma, and research support from Eisai and UCB. K. White is an independent research consultant who was paid by S.M. Mirsattari to assist with the data analysis and editing of the manuscript. S.M. Mirsattari is on the advisory boards and speaker bureaus for UCB Canada Inc., Eisai Limited, and Sunovion Pharmaceuticals Canada, Inc. S.R. Mousavi and B. Hayman-Abello declare that they have no known competing financial interests or personal relationships which have, or could be perceived to have, influenced the work reported in this article.

## Data Availability

Table 1 (Original data) (Mendeley Data).

## References

[1] Massot-Tarrús A., Yu Y.Joe, AlKhateeb M., Mirsattari S.M. (2022 Jun 23). Factors associated with comorbid epilepsy in patients with psychogenic non-epileptic seizures: a large cohort study. Epilepsy. Behav..

[2] Carroll L.J., Cassidy J.D., Holm L., Kraus J., Coronado V.G. (2004 Feb). Methodological issues and research recommendations for mild traumatic brain injury: The WHO Collaborating Centre Task Force on Mild Traumatic Brain Injury. J. Rehabil. Med..

[3] Ruff R.M., Iverson G.L., Barth J.T., Bush S.S., Broshek D.K. (2009 Feb). Recommendations for diagnosing a mild traumatic brain injury: A national academy of neuropsychology education paper. Arch Clin Neuropsychol.

[4] LaFrance W.C., Baker G.A., Duncan R., Goldstein L.H., Reuber M. (2013 Nov). Minimum requirements for the diagnosis of psychogenic nonepileptic seizures: a staged approach. Epilepsia.

